# Comparative Efficacy and Safety of Novel Antiplatelets and Standard Therapy in Patients With Coronary Artery Disease

**DOI:** 10.7759/cureus.71333

**Published:** 2024-10-12

**Authors:** Ravindra Reddy Gangavarapu, Sayed A Mahmud, Anura Manandhar, Ghadeer Sabir, Hala A Abdelhady, Adoum Oumar Abakar, Sondos T Nassar

**Affiliations:** 1 Internal Medicine, California Institute of Behavioral Neurosciences and Psychology, Fairfield, USA; 2 Internal Medicine and Clinical Research, California Institute of Behavioral Neurosciences and Psychology, Fairfield, USA; 3 Medicine and Surgery, Jordan University of Science and Technology, Amman, JOR

**Keywords:** acute coronary syndrome (acs), clopidogrel, coronary artery disease (cad), dual-antiplatelet therapy (dapt), gastrointestinal bleeding (gib), p2y12 antagonists, prasugrel, safety and efficacy, systematic review, ticagrelor

## Abstract

Coronary artery disease (CAD) is a significant health concern that has affected approximately 110 million people worldwide. CAD is defined as persistent narrowing of the coronary arteries as a result of atherosclerotic plaque build-up. Acute coronary syndrome (ACS), which encompasses ST-elevation myocardial infarction (STEMI), non-ST-elevation myocardial infarction (NSTEMI), and unstable angina, often results from plaque ruptures. Platelets are crucial for atherogenesis, vascular inflammation, and oxidative stress. Antiplatelet therapy aimed at reducing thrombotic events is vital for ACS treatment. Clinical guidelines advise the use of dual antiplatelet therapy (DAPT) that combines aspirin and a P2Y12 receptor inhibitor (clopidogrel, prasugrel, or ticagrelor) in ACS patients undergoing percutaneous intervention (PCI). This study aimed to assess comprehensively the effectiveness and safety of ticagrelor and prasugrel in comparison to clopidogrel in patients with ACS. An extensive literature search was conducted using PubMed, PubMed Central (PMC), ScienceDirect, and EBSCO databases. The search revealed studies that compared ticagrelor and prasugrel to clopidogrel in ACS patients, and we selected these studies based on specific inclusion and exclusion criteria, which included observational studies, clinical trials, literature reviews, and meta-analyses involving adult ACS patients treated with ticagrelor, prasugrel, or clopidogrel. The efficacy outcomes were defined as major adverse cardiovascular events (MACE) and thrombotic events, whereas the safety outcomes were measured by major and minor bleeding and hemorrhagic stroke. After a rigorous quality assessment to minimize bias, 23 studies were selected for analysis. The findings indicated that novel antiplatelets reduced MACE but increased bleeding complications, with ticagrelor consistently associated with dyspnea. In conclusion, novel P2Y12 inhibitors provide cardiovascular benefits but require careful patient selection and monitoring due to gastrointestinal bleeding (GIB) risks. Future research should standardize bleeding definitions and assess long-term outcomes. Ticagrelor and prasugrel may be more effective and safer than clopidogrel in ACS patients. Given the high risk of GIB, especially among older individuals or those with a past stroke, it is advisable to suggest a lower prasugrel dose without raising the bleeding rates. Since fewer patients use the novel antiplatelet regimen compared to clopidogrel, future clinical trials should include a broader patient population and compare these regimens.

## Introduction and background

Coronary artery disease (CAD), continues to rank among the foremost sources of mortality and morbidity on a global scale, presenting a substantial public health challenge with an estimated prevalence of more than 110 million cases worldwide [1]. Approximately 20.5 million Americans aged ≥20 years are affected by CAD as per 2024 statistics [2]. It is characterized by a gradual narrowing or blockage of the coronary arteries owing to the accumulation of atherosclerotic deposits. The consequence of this is diminished blood flow to the heart, which can result in myocardial ischemia, infarction, and potentially life-threatening complications such as myocardial infarction (MI) and sudden cardiac death [3]. After decades of gradual progression, the primary factors responsible for the emergence of acute coronary syndrome (ACS) are the rupture of atherosclerotic plaques, which trigger platelet aggregation and subsequent formation of thrombi that result in partial or complete occlusion of the coronary artery. This obstruction can hinder the flow of blood to the heart muscle [4]. The term ACS refers to patients who display acute myocardial ischemia or infarction, which is confirmed or suspected. The three traditional forms of ACS are ST-elevation myocardial infarction (STEMI), non-ST-elevation myocardial infarction (NSTEMI), and unstable angina [5].

**Figure 1:**
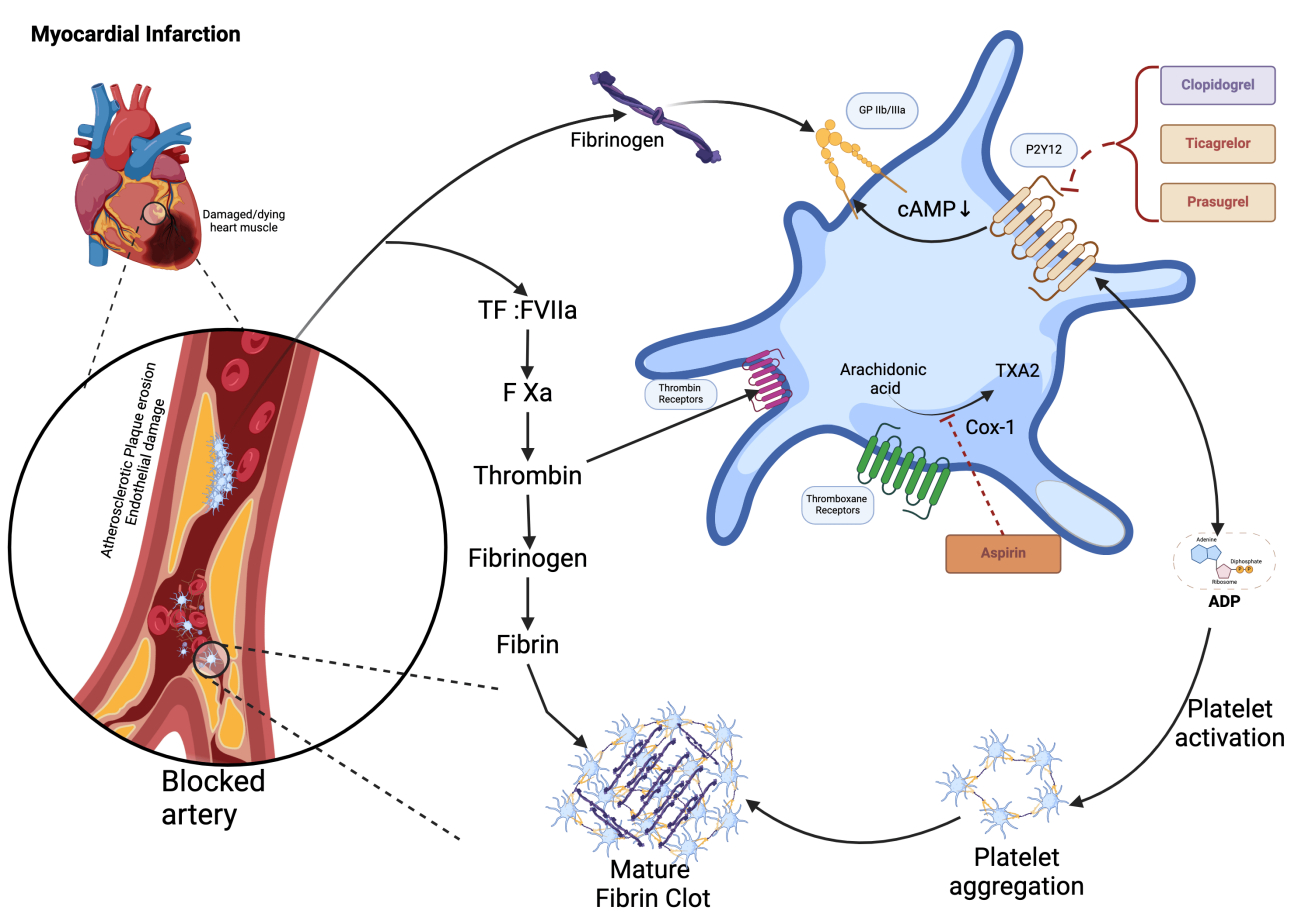
Atherosclerotic plaque and platelet interplay. The image demonstrates the involvement of platelets in atherosclerotic plaque formation and the action of key antiplatelet drugs. The image is created by the authors of this study using BioRender.com.

Platelets hold a crucial position in the development of atherosclerosis and vascular inflammation demonstrated in Figure 1, and a multitude of studies have indicated that platelets are a significant contributor to oxidative stress in ACS [6]. The use of antiplatelet therapy has been a key component in the treatment of ACS, as it aims to prevent the recurrence of thrombotic events by inhibiting platelet activation and aggregation, which is crucial in secondary prevention. Although aspirin has historically been the mainstay of antiplatelet therapy [7], its limitations, including resistance and increased risk of bleeding, have led to the development of new-generation antiplatelet agents with potentially improved efficacy and safety profiles [8].

Clinical guidelines such as the European Society of Cardiology 2023 recommend that patients with ACS undergoing percutaneous coronary stenting (PCI) should start dual antiplatelet therapy (DAPT) that combines aspirin and a P2Y12 receptor inhibitor (clopidogrel, prasugrel, or ticagrelor) to avert the recrudescence of cardiovascular events [7,9]. Clopidogrel, a thienopyridine prodrug, is metabolized into an active form primarily by the hepatic enzyme cytochrome P450 (CYP) 2C19 [10]. Genetic polymorphisms of CYP2C19 may affect a patient's response to clopidogrel. However, it has notable limitations owing to its delayed onset of action, variable antiplatelet effects, and low bioavailability, which can be further influenced by drug interactions, potentially leading to unfavorable clinical consequences. Prasugrel and ticagrelor, new-generation oral inhibitors of the P2Y12 adenosine diphosphate receptor, provide faster and more consistent platelet inhibition than clopidogrel. Clinical trials and recent meta-analyses have demonstrated their superiority in averting ischemic episodes in ACS patients. Furthermore, ticagrelor and prasugrel have shown greater efficacy in inhibiting platelets and improving fibrinolytic capacity, which reduces the likelihood of thrombotic incidents in individuals who have high platelet reactivity while on treatment, when compared to clopidogrel [11].

Novel P2Y12 inhibitors offer several advantages over clopidogrel in controlled settings, as demonstrated in randomized clinical trials (RCTs) like the Trial to Assess Improvement in Therapeutic Outcomes by Optimizing Platelet Inhibition with Prasugrel-Thrombolysis in Myocardial Infarction 38 (TRITON-TIMI 38 ) and PLATelet inhibition and patient Outcomes (PLATO) [12,13]. However, concerns regarding bleeding risk and tolerability have also emerged with these agents, necessitating a comprehensive evaluation of their safety profile compared with standard therapy. However, their comparative effectiveness in real-world scenarios remains less clear [14]. Variability in effectiveness may be due to factors such as the timing of mortality risk post-MI and differences in clinical and patient characteristics, including sex and ACS presentation (STEMI vs. NSTEMI and unstable angina).

Objectives

This systematic review evaluated the effectiveness and safety of novel antiplatelet agents, such as prasugrel and ticagrelor, compared to clopidogrel, in patients with ACS undergoing invasive or non-invasive management. By understanding data from RCTs, observational studies, and meta-analyses, we assessed the impact of various antiplatelet regimens on clinical outcomes such as bleeding complications, major cardiovascular events including myocardial infarction, cardiovascular death, or ischemic stroke, and overall mortality. The results of this study hold considerable consequences for clinical practice, allowing healthcare professionals to choose the most suitable antiplatelet treatments for each patient. Identifying the most effective and safe regimens aims to optimize CAD management, improve patient outcomes, and reduce cardiovascular morbidity and mortality globally.

## Review

Methodology

In this systematic review, we have adhered to the 2020 Preferred Reporting Items for Systematic Reviews and Meta-Analyses (PRISMA) guidelines [15].

Database and Search Strategy

A comprehensive search strategy was developed to locate pertinent studies in various electronic databases which included PubMed, PubMed Central (PMC), ScienceDirect, and EBSCO databases. The most recent search for all databases was conducted in February 2024. The primary search terms utilized in various search engines comprised coronary artery disease, ticagrelor, prasugrel, clopidogrel, and the Medical Subject Heading (MeSH) approach employed in PubMed. The detailed search strategy for each database is presented in Table 1. Table 2 provides additional information on the search strategies and databases.

**Table 1:** Search strategy used to identify relevant studies.

Databases	Keywords	Search strategy	Filters	Search result
PubMed	Ticagrelor or prasugrel, clopidogrel, coronary artery disease	(((((((((((((((((("Coronary Artery Disease"[Mesh]) OR (Artery Disease, Coronary)) OR (Artery Diseases, Coronary)) OR (Coronary Artery Diseases)) OR (Left Main Coronary Artery Disease)) OR (Left Main Disease)) OR (Left Main Diseases)) OR (Left Main Coronary Disease)) OR (Coronary Arteriosclerosis)) OR (Arterioscleroses, Coronary)) OR (Coronary Arterioscleroses)) OR (Atherosclerosis, Coronary)) OR (Atheroscleroses, Coronary)) OR (Coronary Atheroscleroses)) OR (Coronary Atherosclerosis)) OR (Arteriosclerosis, Coronary) OR ( "Coronary Artery Disease/complications"[Majr] OR "Coronary Artery Disease/drug therapy"[Majr] OR "Coronary Artery Disease/mortality"[Majr] OR "Coronary Artery Disease/physiopathology"[Majr] OR "Coronary Artery Disease/therapy"[Majr] )) AND ((((("Ticagrelor"[Mesh]) OR (Brilique)) OR (AZD 6140)) OR (AZD-6140)) OR (Brilinta) OR ( "Ticagrelor/administration and dosage"[Majr] OR "Ticagrelor/adverse effects"[Majr] OR "Ticagrelor/analysis"[Majr] OR "Ticagrelor/pharmacokinetics"[Majr] OR "Ticagrelor/pharmacology"[Majr] OR "Ticagrelor/therapeutic use"[Majr] ) AND ((y_10[Filter]) AND (ffrft[Filter]) AND (humans[Filter]) AND (English[Filter])))) OR ((((((((((((("Prasugrel Hydrochloride"[Mesh]) OR (Hydrochloride, Prasugrel)) OR (Prasugrel HCl)) OR (HCl, Prasugrel)) OR (747, CS)) OR (CS-747)) OR (CS747)) OR (Efient)) OR (Effient)) OR (LY 640315)) OR (640315, LY)) OR (LY640315)) OR (LY-640315) OR ( "Prasugrel Hydrochloride/administration and dosage"[Majr] OR "Prasugrel Hydrochloride/adverse effects"[Majr] OR "Prasugrel Hydrochloride/analysis"[Majr] OR "Prasugrel Hydrochloride/pharmacokinetics"[Majr] OR "Prasugrel Hydrochloride/pharmacology"[Majr] OR "Prasugrel Hydrochloride/therapeutic use"[Majr] ))) AND ((((((((((((((((("Clopidogrel"[Mesh]) OR (SC 25989C)) OR (SC 25990C)) OR (SR 25989)) OR (Clopidogrel-Mepha)) OR (Clopidogrel Mepha)) OR (Clopidogrel Sandoz)) OR (Iscover)) OR (Clopidogrel Napadisilate)) OR (Clopidogrel Hydrochloride)) OR (PCR 4099)) OR (PCR-4099)) OR (Clopidogrel Besylate)) OR (Clopidogrel Besilate)) OR (Clopidogrel, (+)(S)-isomer)) OR (Plavix)) OR (Clopidogrel Bisulfate) OR ( "Clopidogrel/administration and dosage"[Majr] OR "Clopidogrel/adverse effects"[Majr] OR "Clopidogrel/analysis"[Majr] OR "Clopidogrel/pharmacokinetics"[Majr] OR "Clopidogrel/pharmacology"[Majr] OR "Clopidogrel/therapeutic use"[Majr] ))	Free full text, from 2019 to 2024, English, humans	265
PMC	Ticagrelor or prasugrel, clopidogrel, coronary artery disease	"ticagrelor" OR "prasugrel" AND "clopidogrel" AND "coronary artery disease"	5 years cutoff	4107
Science Direct	Ticagrelor or prasugrel, clopidogrel, coronary artery disease	"ticagrelor" OR "prasugrel" AND "clopidogrel" AND "coronary artery disease"	5 years cutoff, English open access, and archive	1837
EBSCO	Ticagrelor or prasugrel, clopidogrel, coronary artery disease	"ticagrelor" OR "prasugrel" AND "clopidogrel" AND "coronary artery disease"	5 years cutoff, full text, English	302

**Table 2:** Inclusion and exclusion criteria of this study. RCTs: randomized control trials

Variables	Inclusion	Exclusion
Research paper	P2Y12 inhibitors and acute coronary syndromes	Non P2Y12 inhibitors
Publication date	Last five years (2019-2024)	Above five years
Literature	Published literature	Author letters, conference abstracts, books, grey literature, non-published literature
Study types and designs	RCT, observational studies, literature review, systematic review, meta-analyses	Case reports
Sample size	Any sample size	-
Population	People with coronary artery disease	Those not affected by coronary artery disease
Sex	Both females and males	-
Age of population	All ages	-
Species	Humans	Animals
Language	English	Other than English
Text availability	Free full text only	Abstracts, paid full texts

Eligibility Criteria

The Population, Intervention, Comparison, Outcome (PICO) framework was utilized to develop this review question, considering the population, intervention, control, and outcome, which includes the population (coronary artery disease (CAD), intervention (newer antiplatelet agents (ticagrelor and prasugrel), control (standard therapy with clopidogrel), and outcomes (efficacy and safety outcomes, such as a reduction in major adverse cardiovascular events {MACE} and bleeding complications). Additional inclusion and exclusion standards are outlined in Table 2.

Selection of Studies

References were systematically arranged and sorted using EndNote citation management software (Philadelphia, PA: Clarivate Plc.). The automated feature of the program in conjunction with manual review was used to eliminate duplicates. Subsequently, preliminary screening of the records was conducted by examining titles and abstracts to eliminate impertinent studies. The articles with full-text availability were retrieved from the remaining records. The retrieved articles were assessed using the appropriate quality appraisal tools to mitigate potential bias.

Risk of Bias in Individual Studies

The entire articles retrieved were assessed for quality and risk of bias using evaluation tools that featured different criteria and passing scores. To be considered acceptable, each tool required a minimum score of 70% outlined in Table 3.

**Table 3:** Quality assessment of each study category. AMSTAR: Assessment of Multiple Systematic Reviews 2; NOS: Newcastle-Ottawa Scale; SANRA-2: Scale for the Assessment of Narrative Review Articles 2

Quality assessment tool	Type of study	Number of items and their scoring system	Tool score	Accepted score >70%	Total assessed studies	Number of accepted studies
AMSTAR [16]	Meta-analyses, systematic review	Eleven items scored as "yes" (1 point), "no" (0 points), "cannot answer" (0 points), "not applicable" (0 points)	11	8	8	8
NOS [17]	Cohort	Eight items scored as yes, no, and not applicable	9	7	14	13
SANRA 2 [18]	Narrative review	Six items scored as 0, 1, and 2	12	9	2	1

Results

Study Selection and Quality Assessment

**Figure 2:**
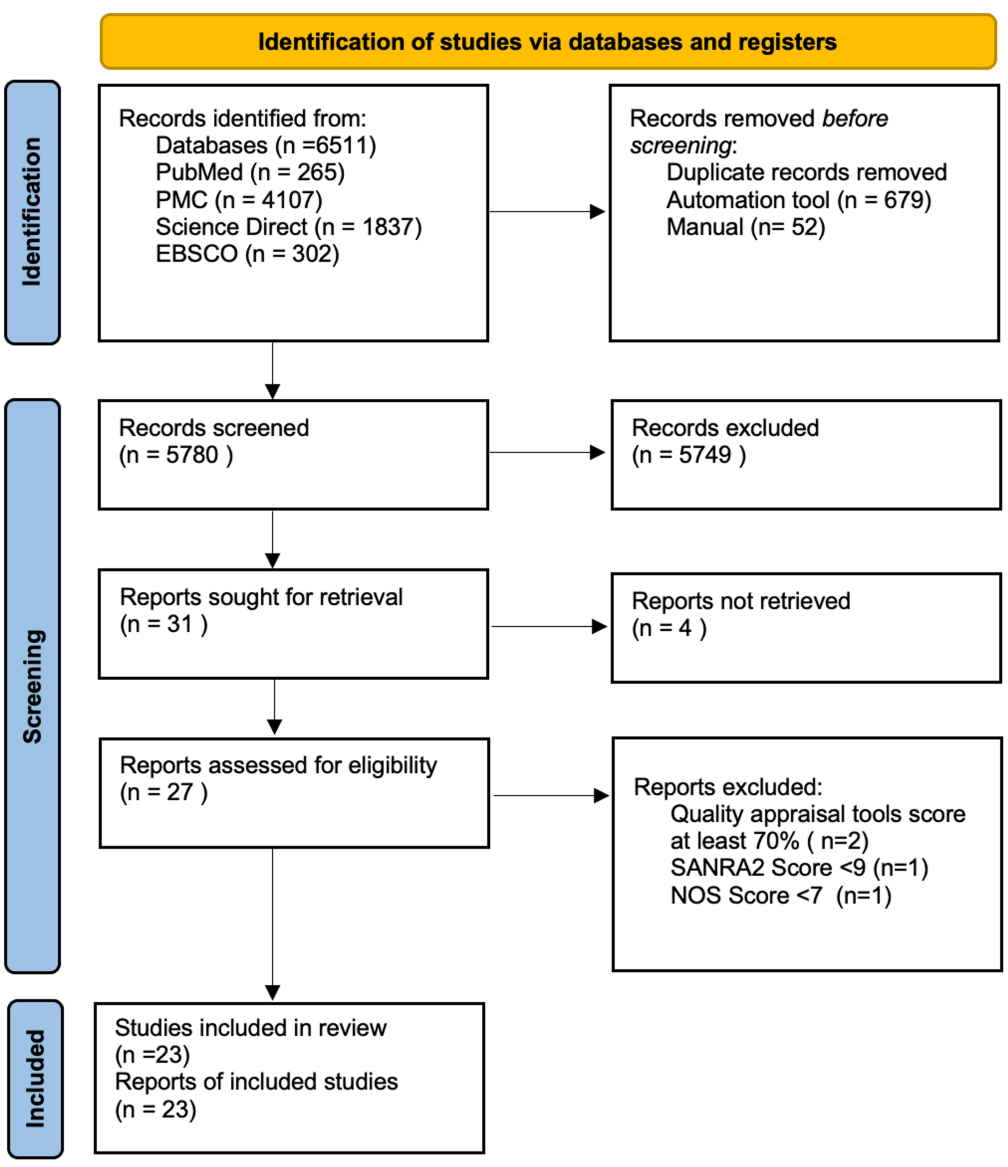
Preferred Reporting Items for Systematic Reviews and Meta-Analyses (PRISMA) flowchart NOS: Newcastle-Ottawa Scale; SANRA-2: Scale for the Assessment of Narrative Review Articles 2

A thorough exploration of PubMed/MEDLINE, PubMed Central (PMC), ScienceDirect, and EBSCO databases yielded 6511 studies. After removing duplicate entries using EndNote, 5780 unique studies remained. Following the title and abstract screening process that adhered to the review's PICO elements and eligibility criteria, 5749 studies were excluded, resulting in the selection of 31 studies for full-text review. The assessment of quality was carried out by the first author, who was subsequently evaluated by the second and third authors. This rigorous process culminated in the selection of 23 studies, including eight meta-analyses, 13 observational studies, one narrative review, and one randomized clinical trial, which scored above 70%. The PRISMA workflow diagram provides a visual representation of the screening and selection procedures shown in Figure 2 [19].

Quality Assessment

Tables 4-8 exhibit the assessment outcomes of each study utilizing the pertinent evaluation tools for each research design, as well as the results of the evaluation process. Table 4 showcases the evaluation scores of systematic reviews and metanalyses using the Assessment of Multiple Systematic Reviews (AMSTER) checklist based on 11 items [16]. Table 5 presents an overview of the NOS assessment tools applicable to observational studies, including prospective and retrospective cohort studies [17]. Table 6 presents a comprehensive analysis of the quality of review articles using the SANRA 2 checklist [18].

**Table 4:** Results of the AMSTER checklist for systematic reviews and meta-analyses by review authors. The passing score was >70%. Responses to the questions in the table were scored as follows: yes (1 point), no (0 points), cannot answer (0 points), and not applicable (0 points). AMSTER: Assessment of Multiple Systematic Reviews

Studies	Was a prior design provided	Was there duplicate selection and data extraction	Was a comprehensive literature search performed	Was the status of publication (e.g., grey literature) used as an inclusion criterion	Was a list of studies (included and excluded provided)	Was the characteristic of the included studies provided	Was the scientific quality of the included studies assessed and documented?	Was the scientific quality of the included studies used appropriately in formulating conclusions?	Were the methods used to combine the findings of studies appropriate	Was the likelihood of publication bias assessed	Was the conflict of interest included	Pass/fail
Farmakis et al. 2022 [20]	Yes	Yes	Yes	Yes	Yes	Yes	Yes	Yes	Yes	Yes	Yes	Pass
Yoon et al. 2020 [10]	No	Yes	Yes	Yes	Yes	Yes	Yes	Yes	Yes	Yes	Yes	Pass
Schreuder et al. 2020 [21]	Yes	Yes	Yes	Yes	Yes	Yes	Yes	Yes	Yes	Yes	Yes	Pass
Fei et al. 2020 [22]	No	Yes	Yes	Yes	Yes	Yes	Yes	Yes	Yes	Yes	No	Pass
Fujisaki et al. 2023 [23]	Yes	Yes	Yes	Yes	Yes	Yes	Yes	Yes	Yes	Yes	Yes	Pass
Boivin-Proul et al. 2023 [24]	Yes	Yes	Yes	Yes	Yes	Yes	Yes	Yes	Yes	Yes	Yes	Pass
Wang et al. 2020 [25]	Yes	Yes	Yes	Yes	Yes	Yes	Yes	Yes	Yes	Yes	Yes	Pass
Guo et al. 2019 [26]	Yes	Yes	Yes	Yes	Yes	Yes	Yes	Yes	Yes	Yes	Yes	Pass

**Table 5:** Results of the NOS assessment tool for observational studies by review authors. The passing score was 7/9. NOS: Newcastle-Ottawa Scale

Studies	Representativeness of the exposed cohort (1 point)	Selection of the non-exposed cohort (1 point)	Ascertainment of exposure (1 point)	Demonstration that the outcome of the interest was not present at the start of the study (1 point)	Comparability of the cohort-based design/analysis (max 2 points)	Assessment of the outcome (1 point)	Was the follow-up long enough for the outcome to occur? (1 point)	Adequacy of follow-up cohorts (1 point)	Pass/fail
Jacobsen et al. 2021 [27]	1	1	1	1	2	1	1	1	Pass
De Luca et al. 2021 [28]	1	1	1	1	2	1	1	1	Pass
Lam et al. 2021 [29]	1	1	1	1	2	1	1	1	Pass
Soueid et al. 2021 [30]	1	1	1	1	2	1	1	1	Pass
Abraham et al. 2020 [31]	1	1	1	1	2	1	1	1	Pass
Laredo et al. 2020 [32]	1	1	1	1	2	1	1	1	Pass
Ruiz-Nodar et al. 2020 [33]	1	1	1	1	2	1	1	1	Pass
Sachdeva et al. 2023 [34]	1	1	1	1	2	1	1	1	Pass
Selhorst et al. 2019 [35]	1	1	1	1	2	1	1	1	Pass
Ng et al. 2022 [36]	1	1	1	1	2	1	1	1	Pass
Yun et al. 2019 [37]	0.5	1	1	1	2	0.5	1	1	Pass
Paszek et al. 2023 [11]	0.5	1	1	1	1	0.5	0.5	1	Fail
Gager et al. 2020 [38]	1	1	1	1	2	1	1	1	Pass
Koshy et al. 2023 [39]	1	1	1	1	2	1	1	1	Pass

**Table 6:** Results of the SANRA 2 checklist for narrative reviews by the review authors. The passing score was 9/12. SANRA 2: Scale for the Assessment of Narrative Review Articles 2

Studies	Does the review justify the importance of the topic being reviewed?	Does the review clearly state its aims or research questions?	Does the review provide a detailed description of the literature search, including databases and search terms?	Does the review reference relevant, high-quality studies and include a balanced discussion of the literature?	Does the review exhibit scientific reasoning, including critical analysis and synthesis of the literature?	Does the review present data appropriately, using tables, figures, and other visual aids as needed?	Sum	Pass
Abubakar et al. 2023 [40]	2	1	0	2	1	1	7	Fail
Pradhan et al. 2022 [41]	2	2	0	2	2	2	10	Pass

**Table 7:** Results of the risk of bias of randomized controlled trials by the review authors. LR: low risk; UN: unclear; HR: high risk

Study	Random sequence generation	Allocation concealment	Selective reporting	Other sources of bias	Blinding of participants and personnel	Blinding of outcome assessment	Incomplete outcome data
Schnorbus et al. 2021 [42]	LR	LR	LR	UN	LR	LR	UN

**Table 8:** Articles selected for this review and their results. RCT: randomized controlled trial; SR: systemic review; MA: meta-analysis; NR: narrative review; TAPT: triple antiplatelet therapy; DAPT: dual antiplatelet therapy; LoF: loss of function; PCI: percutaneous coronary intervention; MACE: major adverse cardiovascular events; MI: myocardial infarction; GIB: gastrointestinal bleeding; CABG: coronary artery bypass grafting; CV: cardiovascular

Studies	Study type	Results
Abraham et al. 2020 [31]	Cohort	Prasugrel reduces gastrointestinal bleeding (GIB) by 36% among STEMI patients, whereas ticagrelor use reduced by 37% compared to clopidogrel in receiving PCI in ACS
Boivin-Proulx et al. 2024 [24]	SR/MA	Ticagrelor-based DAPT in ACS patients treated with PCI may increase MACE risk compared to prasugrel while de-escalating to clopidogrel at 1 month reduces major bleeding risk without increasing thrombotic events. Evidence is insufficient for de-escalating potent P2Y12 inhibitor doses.
De Luca et al. 2021 [28]	Cohort	Prasugrel or ticagrelor has favorable outcomes in clinical practice compared to clopidogrel, without compromising safety.
Farmakis et al. 2022 [20]	SR/MA	For NSTE-ACS patients planned for invasive management, prasugrel is superior to ticagrelor based on moderate evidence, showing better outcomes in composite cardiovascular efficacy, all-cause death, MI, and stent thrombosis. This supports ESC guideline recommendations.
Fei et al. 2020 [22]	SR/MA	Prasugrel and ticagrelor surpass clopidogrel in preventing stent thrombosis and cardiovascular death in ACS patients. Prasugrel significantly reduces MACE and stent thrombosis but raises bleeding risk. Ticagrelor is typically favored for its lower bleeding risk, whereas prasugrel is apt for high ischemic risk patients with minimal bleeding risk.
Fujisaki et al. 2023 [23]	SR/MA	Prasugrel and ticagrelor demonstrate similar efficacy, with prasugrel most likely to reduce primary endpoints like MI, and all-cause death. No significant bleeding differences were noted.
Gager et al. 2020 [38]	Cohort	Ticagrelor and prasugrel outperform clopidogrel in reducing mortality and MACE due to their potent and consistent effects across clinical characteristics.
Guo et al. 2019 [26]	SR/MA	Ticagrelor and prasugrel significantly increase the risk of GIB and non-CABG major bleeding compared to clopidogrel, including higher risks for upper and unspecified GIB. This risk remains elevated in low-risk patients (<75 years old or body weight ≥60 kg).
Jacobsen et al. 2021 [27]	Cohort	In STEMI patients, ticagrelor and prasugrel decrease all-cause mortality, with prasugrel reducing ischemic events at one year without increasing bleeding-related hospitalizations compared to clopidogrel, and no significant differences in effectiveness or safety between prasugrel and ticagrelor.
Koshy et al. 2023 [39]	Cohort	Ticagrelor and prasugrel show similar one-year efficacy and safety compared to clopidogrel in CCS patients receiving PCI.
Lam et al. 2021 [29]	Cohort	In Hong Kong ACS patients, prasugrel and ticagrelor reduced MACE, MI, overall CV death, ischemic stroke, overall mortality, and mortality from one to five years after the index ACS event compared to clopidogrel. They also showed a reduced bleeding risk over the same period. Overall, prasugrel and ticagrelor offer better clinical benefits than clopidogrel in DAPT for ACS patients.
Laredo et al. 2020 [32]	Cohort	About one in three patients experienced a gastrointestinal event during the first year of DAPT, with similar risk between clopidogrel and newer antiplatelet agents after adjusting for confounding factors. Anemia and iron deficiency were common adverse events often overlooked. Death during DAPT primarily resulted from ischemic cardiovascular issues rather than gastrointestinal bleeding, yet bleeding risk influences the choice between clopidogrel and newer agents.
Ng et al. 2022 [36]	Cohort	Prasugrel and ticagrelor were linked to a lower adjusted risk of ischemic stroke and thrombotic events compared to clopidogrel. The risks of intracranial hemorrhage and major bleeding were similar between the two treatments.
Pradhan et al. 2022 [41]	NR	Prasugrel and ticagrelor are now preferred over clopidogrel for their effectiveness. However, new P2Y12 inhibitors have not yet definitively replaced clopidogrel, and bleeding risks remain a concern. Current practice involves choosing the P2Y12 inhibitor based on the patient’s clinical scenario.
Ruiz-Nodar et al. 2020 [33]	Cohort	Prasugrel and ticagrelor were more effective than clopidogrel in reducing adverse events, with novel P2Y12 inhibitors linked to lower all-cause mortality and MACE without increasing bleeding events. Despite this, clopidogrel remained the most prescribed P2Y12 inhibitor, especially for older and high-risk patients.
Sachdeva et al. 2023 [34]	Cohort	Ticagrelor was associated with lower all-cause mortality compared to clopidogrel but similar rates of MI, stroke, and bleeding. Prasugrel showed similar rates for all studied outcomes compared to clopidogrel. There was also more switching of therapy and less persistence in patients initially on novel P2Y12 inhibitors.
Schnorbus et al. 2021 [42]	RCT	There are significant benefits of prasugrel and ticagrelor over clopidogrel in ACS and high-risk stable patients. Ticagrelor improves microvascular function when administered pre-PCI, but this effect is not maintained in the short term.
Schreuder et al. 2020 [21]	SR/MA	No significant sex differences in the efficacy and safety of prasugrel and ticagrelor were found, indicating that men and women should be treated equally. Sex-specific recommendations for high-potency DAPT are unwarranted and should lead to the adoption of guideline-recommended DAPT for both genders.
Selhorst et al. 2019 [35]	Cohort	Prasugrel and ticagrelor are superior to clopidogrel, particularly with lower rates of high platelet reactivity. However, low platelet reactivity was more common with prasugrel than ticagrelor or clopidogrel. Ticagrelor provides a consistent antiplatelet effect across a wide range of individual platelet responses.
Soueid et al. 2021 [30]	Cohort	Ticagrelor is used for rapid periprocedural platelet inhibition during PCI, followed by a switch to clopidogrel before discharge to improve long-term therapy affordability and compliance. This approach was safe with regard to bleeding, reinfarction, stent thrombosis, and 30-day mortality.
Wang et al. 2020 [25]	SR/MA	A TAPT regimen using prasugrel or ticagrelor with aspirin and GPI may significantly reduce the risk of MACEs without increasing bleeding risk in STEMI patients undergoing primary PCI, compared to clopidogrel-based TAPT.
Yoon et al. 2020 [10]	SR/MA	Alternative antiplatelet treatments based on genotyping can improve clinical outcomes in LoF allele carriers. However, this approach should be tailored to balance the patient's ischemic and bleeding risks.
Yun et al. 2019 [37]	Cohort	Ticagrelor increased bleeding rates but significantly reduced all-cause death, cardiovascular death, and stroke compared to clopidogrel. Prasugrel also increased bleeding without improving effectiveness outcomes. No significant differences were observed between prasugrel and ticagrelor.

Table 7 presents a summary of the risk of bias for the only randomized control trial included in this review [42,43]. Table 8 displays the articles included in this review along with their respective findings.

Discussion

**Figure 3:**
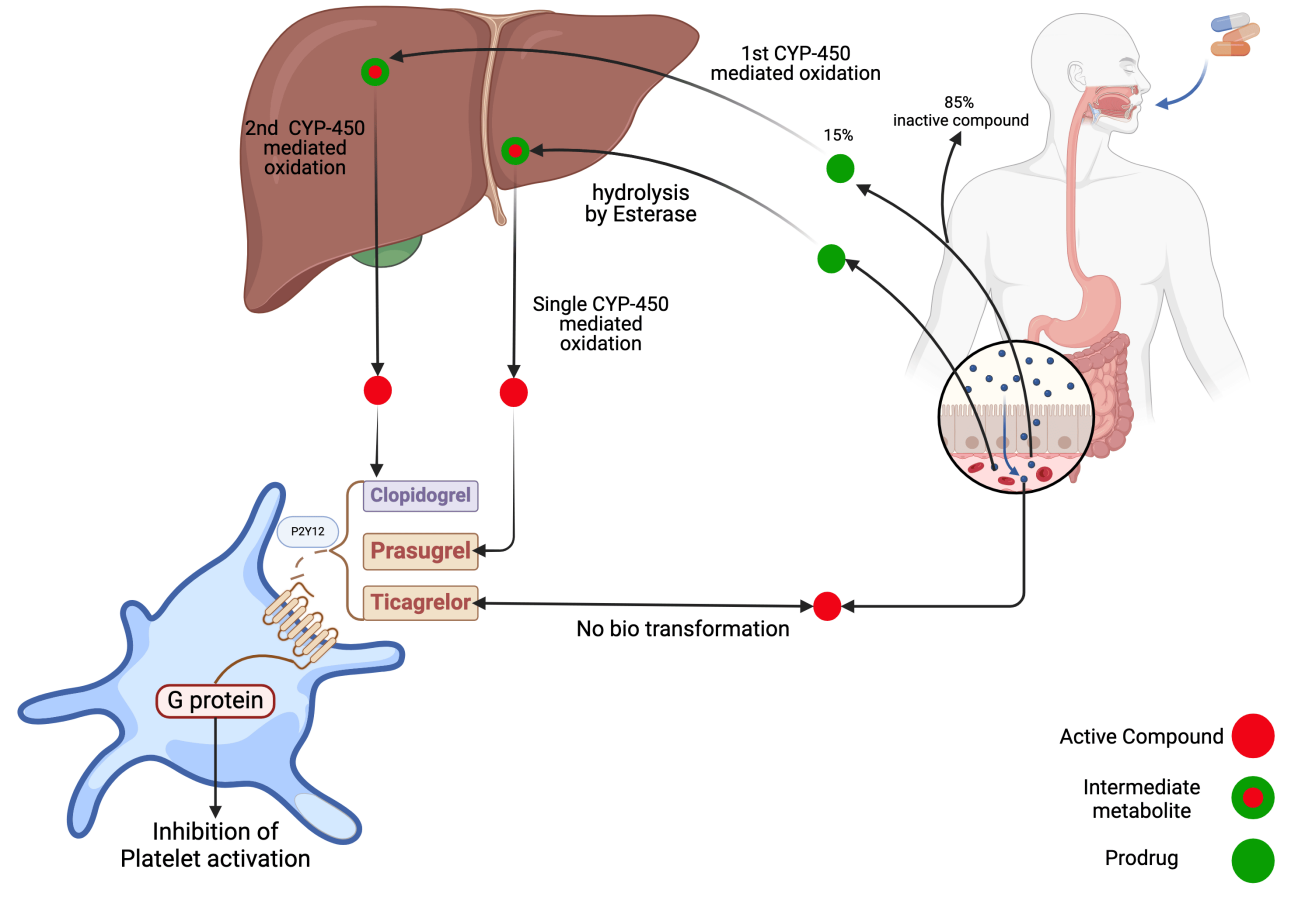
Biotransformation and mechanism of action of clopidogrel, ticagrelor, and prasugrel. Clopidogrel undergoes extensive CYP-mediated oxidation and a very small quantity reaches into final circulation. Ticagrelor is rapidly absorbed in the intestine and does not require any biotransformation. The image is created by the authors of this study using BioRender.com.

Despite advancements in treatment options, CAD still poses a substantial challenge to healthcare systems and society at large, emphasizing the need for efficient therapeutic interventions. The introduction of novel antiplatelet agents, such as P2Y12 receptor inhibitors (e.g., prasugrel and ticagrelor), has generated considerable interest in evaluating their comparative effectiveness and safety in individuals with ACS. These agents possess various mechanisms of action ranging from irreversible inhibition of the P2Y12 receptor (clopidogrel and prasugrel) to direct antagonism (ticagrelor) of platelet activation pathways, providing clinicians with a broader range of options for tailored antiplatelet therapy [40]. Clopidogrel efficacy varies among individuals due to the need for activation by the liver enzyme CYP450. Genetic variations such as CYP450 polymorphisms, particularly CYP2C19 variants, can influence clopidogrel metabolism. Figure 3 illustrates the variations in metabolism associated with distinct P2Y12 inhibitors. Individuals with a slow-metabolizing version of CYP450 may not receive sufficient benefits from clopidogrel, leading to a higher risk of recurrent heart attack or stroke. Conversely, individuals who possess a fast-metabolizing variant may be at a higher risk for bleeding due to excessive activation of the medication [10,40]. This systematic review aimed to compare the efficacy and safety of new-generation prasugrel and ticagrelor with those of standard clopidogrel therapy in patients with CAD by assessing outcomes such as recurrent ischemic events, bleeding complications, and mortality to evaluate the overall impact of novel antiplatelet therapy on patient health.

Risk of Gastrointestinal Bleeding and Other Bleeding Complications

**Figure 4:**
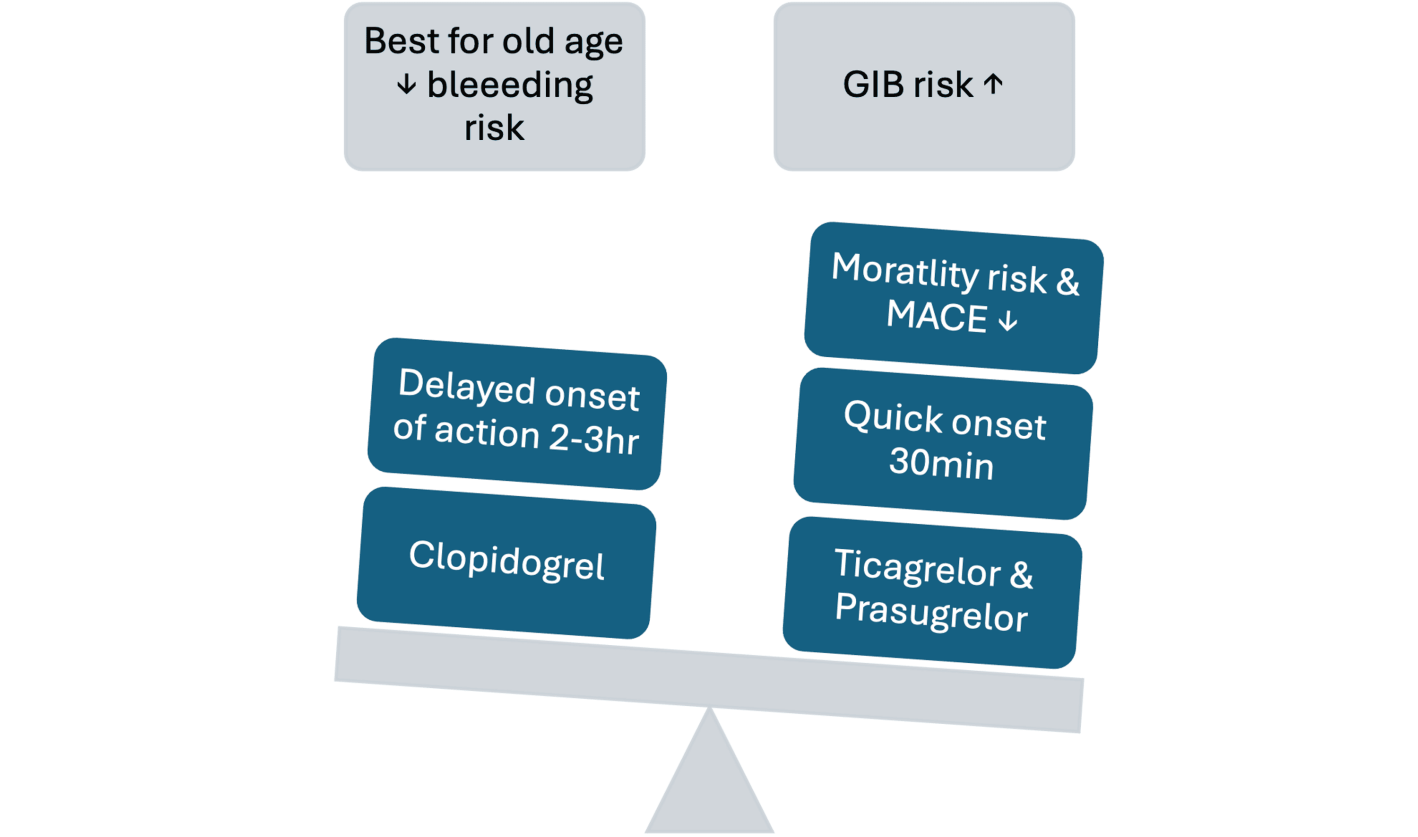
Careful balance is crucial when considering the benefits of novel P2Y12 inhibitors compared to clopidogrel. The image is created by the author (Ravindra Reddy Gangavarapu) of this study.

Gastrointestinal bleeding (GIB) is a significant adverse event that typically happens in the first year after undergoing percutaneous coronary intervention (PCI), often requiring cessation of antiplatelet therapy until the bleeding source is managed. Clinical trials, such as TRITON-TIMI 38 and PLATO, have consistently found the gastrointestinal tract to be the primary location of significant bleeding in individuals receiving treatment with ticagrelor or prasugrel [12,13]. Novel P2Y12 inhibitors posed a higher GIB risk compared to clopidogrel, with a relative risk (RR) of 1.28 (95% CI: 1.13-1.46). Subgroup analyses indicated an increased risk of upper GIB (RR: 1.32, 95% CI: 1.05-1.67) and unspecified GIB (RR: 1.25, 95% CI: 1.01-1.53) with novel P2Y12 inhibitors, as found by Guo et al. [26]. This elevated risk has also been observed in real-world settings, beyond RCTs. Additionally, switching to ticagrelor did not significantly improve bleeding or ischemic outcomes compared to continuing clopidogrel treatment. Due to the increased risk of bleeding in individuals aged 75 years or older, weighing less than 60 kilograms, or with a history of transient ischemic attack or stroke, it is recommended to use a lower prasugrel dose, without increasing the bleeding rate. However, the study had the following limitations: GIB was underreported compared to all major non-coronary artery bypass grafting (CABG) bleeding events, converting different study definitions to the PLATO criteria without original data could introduce bias, and dosage variability among the included studies may affect bleeding outcomes, increasing result variability [13]. This study found that compared to clopidogrel, ticagrelor or prasugrel has been shown to decrease the likelihood of dying and experiencing mortality and major adverse cardiovascular events (MACE) in ACS patients [26]. However, it is essential to balance these cardiovascular benefits with the bleeding risks. This balance is demonstrated in Figure 4.

A cohort study by Laredo et al. uncovered that the disparity in bleeding risk between DAPT regimens comprising prasugrel or ticagrelor and those containing clopidogrel is negligible [29]. This is attributed to varying baseline characteristics as follows: younger patients are more often in the prasugrel group, whereas older or less healthy cardiovascular patients receive clopidogrel. Despite numerous studies highlighting the superior efficacy of modern antiplatelet agents, data on gastrointestinal (GIB) events related to bleeding remain limited. This study highlights the importance of GIB, as almost one-fourth of patients on DAPT (29.5%) experience at least one GIB event, predominantly anemia, which can impair cardiovascular health. Managing bleeding with DAPT is complex and requires a balance between bleeding and ischemia risk. Many patients resume DAPT after a bleeding event and continue taking proton pump inhibitors (PPIs) beyond the recommended period following the guidelines. It is essential not to restrict novel antiplatelet therapy to individuals with a low likelihood of GIB, as there is no significant danger associated with it, despite the common perception that such therapy is only beneficial for high-risk patients. Despite the large sample size, the study had limitations, including baseline characteristic differences in major GIB between the groups and varied bleeding definitions. Additionally, the retrospective nature of this study may have introduced residual confounders. However, the main advantage of this study is its focus on GIB, comprehensive endoscopic evaluations, and detailed reporting of anemia, which is a significant but often overlooked adverse event.

Boivin-Proulx et al.'s meta-analysis indicated that ticagrelor had a higher incidence of MACE than prasugrel, although the evidence was of low certainty. Conversely, shifting from a potent P2Y12 inhibitor to clopidogrel after one month resulted in a decrease in major bleeding events while maintaining a stable rate of thrombotic events in ACS patients following PCI [24]. Compared to clopidogrel, ticagrelor reduced GIB by 37% across all subgroups (NSTE-ACS and STEMI), whereas prasugrel reduced GIB by 21% in STEMI patients. There were no differences between ticagrelor and prasugrel in a large, diverse national cohort [31]. However, observational studies in real-world settings may have unmeasured confounding variables.

A real-world community-based study by Sachdeva et al. found that when comparing ticagrelor and clopidogrel in ACS patients undergoing PCI, ticagrelor was linked to lower all-cause mortality than clopidogrel after adjusting for multiple variables and propensity, with similar rates of myocardial infarction, stroke, and bleeding between the two groups. Prasugrel had outcomes similar to those of clopidogrel across the significant measures. This study also noted higher rates of therapy switching and lower treatment persistence among patients initially treated with novel P2Y12 inhibitors. Further randomized studies reflecting real-world populations are required to confirm these findings [34].

A retrospective observational study conducted by Laredo et al. revealed that approximately one-third of the patients encountered a gastrointestinal event within the initial 12 months of DAPT administration, highlighting its prevalence. After taking into account confounding variables, the risk of gastrointestinal issues was found to be comparable between patients who were administered clopidogrel and those who received newer antiplatelet DAPT. Anemia and iron deficiency are the most prevalent adverse events, yet they are frequently disregarded in many studies. Although ischemic cardiovascular events are the leading causes of death during DAPT, gastrointestinal bleeding is less significant. Clinicians usually choose between clopidogrel and newer agents based on bleeding risk. Further research should explore the indications and cardiovascular benefits of new antiplatelet agents in a broader patient population [32].

Other Bleeding Complications

Atherosclerotic inflammation caused by ACS can result in recurrent atherothrombotic events, including stroke. Post-ACS stroke is particularly pernicious, with up to one-third of patients dying and over two-thirds of survivors experiencing poor functional outcomes. In the standardized composite endpoint definition by the Academic Research Consortium, stroke is the second most critical endpoint. Ng et al. found that in patients with ACS from China who received their initial PCI in Hong Kong, potent P2Y12 inhibitors were linked to a reduced adjusted risk of ischemic stroke and thrombotic events compared to clopidogrel, with similar rates of intracranial hemorrhage (ICH) and major bleeding events between the two groups [36]. Major bleeding, as per the International Society on Thrombosis and Hemostasis, includes instances of severe bleeding such as intracranial, intraarticular, intramuscular (with compartment syndrome), intraocular, pericardial, and retroperitoneal bleeding, as well as cases requiring transfusions or resulting in a decrease in hemoglobin of 20 g/L or more. Furthermore, studies on all-cause major bleeding unrelated to CABG indicate a greater risk associated with the use of novel P2Y12 inhibitors, as noted by Guo et al [26]. Prasugrel was found to be correlated with an elevated likelihood of deadly bleeding in a fixed-effect model (RR: 2.03, 95% CI: 1.10-3.72), with intracranial bleeding being the predominant location for lethal bleeding, although the results were not statistically significant when using a random effect model (RR: 1.92, 95% CI: 0.69-5.56).

Switch Strategy

Soueid et al. undertook a retrospective observational study comprising 5007 patients (average age of 63.5 years, with a standard deviation of 12.5 years), finding that 54.8%, 8.5%, and 36.7% were preloaded with ticagrelor, prasugrel, and clopidogrel, respectively, before PCI. Notably, 93% of the ticagrelor-loaded patients and 58% of the prasugrel-loaded patients were switched to clopidogrel for long-term therapy before discharge. This switching strategy resulted in the lowest bleeding rates (0.9% for ticagrelor and 0.8% for prasugrel) compared with those maintained on ticagrelor (2.5%) or clopidogrel (1.7%). This study suggests that this approach is safe and effective, warranting further exploration of the impact of increased adherence to more affordable clopidogrel [30]. Boivin-Proulx et al.'s meta-analysis showed that in ACS patients who underwent PCI, the use of ticagrelor in the DAPT regimen was associated with a greater risk of MACE compared to prasugrel, based on low-certainty evidence. Switching a potent P2Y12 inhibitor to clopidogrel after one month can minimize the risk of major bleeding without raising the chances of developing thrombotic events. However, more evidence is needed to determine the efficacy and safety of reducing the dose of potent P2Y12 inhibitors in ACS patients initially treated with a full-dose potent P2Y12 inhibitor [24].

Platelet Reactivity

A randomized, blinded parallel group trial by Schnorbus et al. unveiled that microvascular function in individuals who underwent PCI for ACS substantially improved two hours after administering prasugrel post-procedure with less improvement observed with ticagrelor. This effect disappeared after one day and was absent at the 30-day mark, both immediately after the stent procedure and in subsequent weeks [42]. Selhorst et al.'s retrospective observational study emphasized balancing platelet inhibition with reducing ischemic and bleeding complications in ACS patients. Emerging strategies suggest that potent platelet inhibitors are the most beneficial shortly after ACS, with subsequent de-escalation to less potent therapies to prevent bleeding. Personalized antiplatelet therapy faces challenges because guidelines do not recommend routine platelet function tests, and there is insufficient clinical data supporting personalized therapy for dosage adjustments or P2Y12 inhibitor strategy changes. This evaluation concluded that prasugrel and ticagrelor significantly reduced high-on-treatment platelet reactivity (HPR) compared to clopidogrel. Low-on-treatment platelet reactivity (LPR) was found to be more prevalent in individuals taking prasugrel compared to those taking ticagrelor or clopidogrel. Ticagrelor offered consistent antiplatelet effects with the broadest range of platelet reactivity inhibition, as shown by the highest normalized coefficient of variation. The observational nature of this study is a significant limitation, necessitating more randomized prospective studies to guide antiplatelet therapy in patients with ACS [35]. A post hoc analysis of three prior observational studies by Ranucci et al. revealed that women generally have a higher platelet count than men, which is linked to increased platelet reactivity. It is noteworthy that women with elevated platelet counts are more likely to experience high platelet reactivity (HPTR), implying that women who require P2Y12 inhibitors might gain an advantage from higher drug levels or antiplatelet agents with a lower incidence of HPTR [44].

STEMI-ACS

Jacobsen et al. found that in patients with STEMI who received PCI, both ticagrelor and prasugrel were found to decrease all-cause mortality, with prasugrel showing a greater decrease in ischemic events without increasing hospitalization-required bleeding than clopidogrel. No notable distinctions in effectiveness or safety were observed between ticagrelor and prasugrel [27].

Wang et al.'s meta-analysis indicated that a TAPT regimen comprising ticagrelor or prasugrel, in conjunction with aspirin and a GPI, could considerably decrease the risk of MACEs in STEMI patients receiving PCI without increasing the bleeding risk, as compared to a clopidogrel-containing TAPT regimen. However, additional confirmation through a large-scale RCT with sufficient sample size and prolonged follow-up is necessary [25].

De Luca et al.'s propensity score-matched observational study indicated that new P2Y12 inhibitors have demonstrated a substantial reduction in cardiovascular death and stroke when compared to clopidogrel. Although the major bleeding rates were numerically similar to the all-cause mortality differences, they failed to show statistical significance. No disparities in ischemic or bleeding outcomes were observed between the prasugrel and ticagrelor groups, suggesting that both have better clinical outcomes than clopidogrel while maintaining a similar safety profile [28].

NSTE-ACS

According to Fujisaki et al., potent P2Y12 inhibitors can significantly improve the prognoses of patients with NSTE-ACS owing to their reliable pharmacological properties. The P score evaluation revealed that prasugrel was the efficient treatment for all ischemic outcomes, except for cardiovascular death. In comparison to clopidogrel, prasugrel demonstrated a lower incidence of MACE (HR: 0.84; 95% CI: 0.71-0.99) and cardiac infarction (high risk {HR}: 0.82; 95% CI: 0.68-0.99), with no significant increase in major bleeding risk (HR: 1.30; 95% CI: 0.97-1.74). On the other hand, ticagrelor decreased the probability of cardiovascular mortality (HR: 0.79; 95% CI: 0.66-0.94) but elevated the likelihood of major bleeding (HR: 1.33; 95% CI: 1.00-1.77; P=0.049) relative to clopidogrel. Prasugrel demonstrated the highest likelihood of MACE reduction (P=0.97), surpassing ticagrelor (P=0.29) and clopidogrel (P=0.24). Additional studies are necessary to identify the most effective P2Y12 inhibitors for patients with NSTE-ACS, including large-scale, multi-ethnic clinical trials and research on novel DAPT approaches [23].

Farmakis et al. reported that prasugrel outperformed ticagrelor in patients with NSTE-ACS undergoing invasive management, significantly reducing the primary efficacy endpoint compared with clopidogrel (HR: 0.81, 95% CI: 0.67-0.99), whereas ticagrelor showed no considerable difference (HR: 1.01, 95% CI: 0.79-1.29). Direct comparisons indicated better outcomes with prasugrel than with ticagrelor (HR: 0.80, 95% CI: 0.61 to 1.06), although the disparity was not statistically substantial. Prasugrel ranked highest in efficacy (P=0.96) compared to clopidogrel (P=0.28) and ticagrelor (P=0.26), significantly reducing the composite cardiovascular endpoint (HR: 0.76, 95% CI: 0.61-0.95) and ticagrelor (HR: 0.74, 95% CI: 0.56-0.98). Ticagrelor markedly decreased cardiovascular death relative to clopidogrel (HR: 0.81, 95% CI: 0.69-0.96), while prasugrel did not (HR: 0.91, 95% CI: 0.80-1.04). Prasugrel use resulted in lower rates of composite cardiovascular outcomes, all-cause mortality, myocardial infarction, and definite stent thrombosis. It was identified as the most effective P2Y12 inhibitor, consistent with ESC guidelines [45], which recommend it over ticagrelor for invasively managed patients with NSTE-ACS. Despite criticism of key studies [46], the Intracoronary Stenting and Antithrombotic Regimen: Rapid Early Action for Coronary Treatment (ISAR-REACT 5) trial, which enrolled a substantial number of patients with NSTE-ACS, faced criticism for its open-label design [47]. The PLATO trial was criticized for including conservatively managed patients [13], whereas the TRITON-TIMI 38 study focused on PCI as the first-line treatment [12]. Reranking the treatments without considering the results of the ISAR-REACT 5 sensitivity analysis did not result in any alterations to the order of the treatments, confirming the efficacy of prasugrel [20]. According to the available evidence, prasugrel appears to offer a clinical edge in minimizing ischemic events and fatalities, while concurrently maintaining a consistent profile of major bleeding risks when contrasted with clopidogrel, reinforcing guideline recommendations for its use, as noted by Farmakis et al. and Pradhan et al. [20,41].

Zhang et al. reported that third-generation oral P2Y12 inhibitors, specifically ticagrelor, were linked to a higher incidence of dyspnea (relative risk {RR}: 2.15, 95% confidence interval {CI}: 1.59-2.92), unlike prasugrel (RR: 1.03, 95% CI: 0.86-1.22) [48]. A cohort study by Yun et al. on East Asian patients with ACS found that ticagrelor, compared to clopidogrel, led to increased bleeding, but significantly reduced overall mortality, cardiovascular deaths, and stroke. Prasugrel, compared with clopidogrel, increased bleeding events without differences in efficacy. No notable distinctions were detected between ticagrelor and prasugrel regarding bleeding or ischemic incidents [37].

Efficacy and Safety

Pretreatment considerations such as administering P2Y12 inhibitors before angiography are not typically recommended due to the heightened risk of bleeding without any substantial ischemic advantages, as demonstrated by the findings of the ACCOAST trial [46]. According to Fei et al., both prasugrel and ticagrelor are more effective than clopidogrel in protecting patients with ACS from stent thrombosis and cardiovascular death, with prasugrel notably reducing MACE, MI, and stent thrombosis but increasing bleeding risks TRITON-TIMI 38 trial. Personalized ACS treatment must consider each patient's benefit-risk profile, with ticagrelor often preferred owing to its favorable safety profile. Prasugrel should not be administered to individuals who have a higher likelihood of bleeding, such as those with a past record of stroke, TIA, surgery, trauma, falls, cancer, age ≥75 years, or weighing <60 kg, but it is suggested for ACS patients who have a high risk of ischemia and a low risk of bleeding. The POPular AGE trial highlighted the importance of considering bleeding risk in elderly patients with NSTE-ACS. Although observational studies have reported inconsistent results and are subject to bias, they are valuable for translating RCT findings to clinical practice settings for the general population [22].

Ruiz-Nodar et al. found that in the ACS registry, which reflects current practice, novel P2Y12 inhibitors such as prasugrel and ticagrelor were more effective in reducing adverse events than clopidogrel ACS individuals. Prescribing these novel inhibitors at the time of discharge was found to be autonomously associated with a lower risk of all-cause mortality and MACE, without an increased incidence of bleeding. Despite this, clopidogrel remains the most commonly prescribed P2Y12 inhibitor, especially for older and elevated-risk patients. The addition of ticagrelor to aspirin reduced cardiovascular death and coronary heart disease by 22% and 34%, respectively, in patients with diabetes, emphasizing the importance of potent P2Y12 inhibitors in patients with elevated cardiovascular risk. Low compliance with the guidelines for antiplatelet medication administration upon discharge is a concern, as is the premature discontinuation of these drugs after coronary disease and PCI, which is linked to a higher likelihood of stent thrombosis, recurrent myocardial infarction, ischemic stroke, and cardiac death. Therefore, maintenance of antiplatelet therapy is crucial for achieving optimal outcomes. The underutilization of novel P2Y12 inhibitors in elderly patients may be due to a lack of robust evidence regarding their risk-benefit profile in this significant subgroup and substantial variation among elderly patients. In the registry, patients receiving prasugrel or ticagrelor showed no differences in major bleeding rates compared to those treated with clopidogrel, consistent with both TIMI and Bleeding Academic Research Consortium (BARC) bleeding definitions, and similar results were observed in patients with diabetes [33].

Lam et al. found that in Hong Kong patients with ACS, prasugrel and ticagrelor were more effective than clopidogrel in reducing the risk of MACE and MI up to five years after the ACS event. These newer P2Y12 receptor antagonists have also been linked to lower risks of cardiovascular death, ischemic stroke, and overall mortality during the same period. Additionally, prasugrel and ticagrelor showed a reduced bleeding risk compared to clopidogrel from one to five years post-ACS. Thus, as part of dual antiplatelet therapy (DAPT), prasugrel and ticagrelor offered greater clinical benefits for ACS patients in Hong Kong [29].

Examining the Impact of Sex-Based Bias on Antiplatelet Drug Efficacy

Across all age groups, males demonstrated a greater prevalence of coronary heart disease (CHD) in comparison to females​ [2]. Schreuder et al.'s meta-analysis showed that sex-specific recommendations for potent dual antiplatelet therapy (DAPT) are unnecessary, as there are no significant sex differences in the efficacy and safety of high-potency P2Y12 inhibitors. Women experience worse cardiovascular outcomes than men after acute coronary syndrome (ACS) owing to comorbidities and older age at onset. Discrepancies in ACS management also contribute, with women being less likely to receive recommended treatment, such as reperfusion therapy for STEMI (Swedish Web-System for Enhancement and Development of Evidence-Based Care in Heart Disease Evaluated According to Recommended Therapies {SWEDEHEART} registry). Women receive DAPT less frequently, and when prescribed, clopidogrel is often chosen over the more effective prasugrel, primarily because of perceived higher bleeding risks, although only access site hematomas are more common in women. Further research is needed to reduce bleeding; however, the less aggressive DAPT approach is unjustified. The ADAPT-DES study disclosed that both men and women displaying high platelet reactivity (HPR) faced an elevated risk of stent thrombosis. However, women with HPR were the only ones who experienced a substantial reduction in bleeding risks. HPR was more common in women (51.7% vs. 39.6%; P<0.0001), potentially explaining the sex disparities in clopidogrel response, which is linked to elevated platelet reactivity. Previous research in a laboratory setting has indicated that women exhibit greater platelet responsiveness than men; however, the underlying reason remains undiscovered. Increased levels of estrogen in women may promote platelet clustering, platelet adhesion to fibrinogen, and platelet-leukocyte interactions [21].

Genotype-guided Antiplatelet Therapy

Yoon et al. suggested that genotyping-guided alternative antiplatelet therapies may offer improved clinical outcomes for individuals with the loss-of-function (LoF) allele compared to clopidogrel. These treatments should be customized to the patient's ischemic and bleeding risk. In patients with CAD who possess CYP2C19 LoF alleles, these alternatives demonstrated superior clinical effectiveness compared to clopidogrel, reducing the possibility of MACE without markedly increasing the risk of bleeding. Although many patients with CAD benefit from clopidogrel, their pharmacogenetic limitations lead to recurrent cardiovascular events. The CYP2C19 genotype notably affects adverse cardiovascular outcomes, especially among Asians, who have a higher prevalence of the LoF allele than Caucasians and Africans do. The prevalence of reduced metabolizers (RMs) also varies by ethnicity, from 3-5% in Caucasians to 12-23% in Asians, affecting clopidogrel response. Despite these limitations, this study strongly supports the use of clopidogrel or other alternatives for treating CYP2C19 LoF carriers [10].

Long-Term Benefits of Novel P2Y12 Inhibitors

A study by Gager et al. demonstrated that 2% of patients experience stent thrombosis within a year of undergoing PCI, the incidence of the highest occurrence was observed in the clopidogrel group as compared to the ticagrelor or prasugrel group (4% vs. 1.4% vs. 0.6%; P<0.001). Clopidogrel's efficacy decreases with increasing ACS severity (STEMI > NSTEMI > unstable angina). Elderly STEMI patients (≥75 years) had a diminished risk of MACE within a year of treatment with ticagrelor than clopidogrel (HR: 0.69; 95% CI: 0.49-0.97; P=0.03). Age was the primary predictor of long-term mortality, followed by the type of P2Y12 inhibitor used, highlighting the importance of these medications in long-term consequences. Unlike the PLATO and TRITON-TIMI 38 trials, which had median surveillance periods of nine and 15 months, correspondingly, this study had a median follow-up of 5.6 years, enabling a novel analysis of extended-period mortality rates among individuals treated with clopidogrel, ticagrelor, and prasugrel. Long-term data confirmed prasugrel's and ticagrelor's supremacy over clopidogrel in MACE and mortality, likely due to their strong and consistent effects, regardless of clinical characteristics. The results corroborate the existing ESC recommendations, advocating clopidogrel for stable CAD and ticagrelor or prasugrel for ACS [38].

Chronic Coronary Syndrome

Paszek et al. found that treating patients with advanced chronic coronary syndrome (CCS) with ticagrelor or prasugrel plus aspirin proved to be more effective in reducing platelet activation and plasminogen activator inhibitor-1 (PAI-1) levels compared to aspirin alone or aspirin with clopidogrel. This study indicated that P2Y12 receptor inhibition enhances fibrinolytic capacity and lowers thrombotic risk [11]. Research conducted by Koshy et al. revealed that ticagrelor and prasugrel are becoming increasingly utilized in CCS patients who are undergoing PCI, offering similar effectiveness to clopidogrel over one year. Further research is needed to assess whether these potent P2Y12 inhibitors benefit high-thrombosis, low-bleeding-risk CCS patients undergoing PCI compared to clopidogrel [39].

Strength and limitations

This systematic review undertakes a comprehensive examination of the consequences of novel P2Y12 inhibitors in contrast to clopidogrel in individuals with coronary artery disease. The strengths of this review include meticulous methodology, rigorous quality assessment, and transparent reporting. It employs an extensive search strategy across numerous significant databases by incorporating both MeSH terms and keywords, which capture a wide range of studies. Detailed documentation of the search strategy enhanced transparency and allowed replication. Rigorous inclusion and exclusion criteria were set to ensure the eligibility and relevance of studies, focusing on patients with coronary artery disease, novel P2Y12 inhibitors, comparison with clopidogrel, and efficacy and safety outcomes. This approach minimizes bias and ensures applicability to the research questions. Quality assessment was performed using the AMSTER checklist for systematic reviews and meta-analyses, the Newcastle-Ottawa Scale for observational studies, the risk of bias tool for RCTs, and the SANRA-2 checklist for narrative reviews. Research studies that had a significant risk of bias were not considered, which helped improve the dependability of the overall evidence. Adherence to the PRISMA 2020 guidelines further improves transparency and reporting quality. The review meticulously documents its inclusion/exclusion criteria, search strategy, and quality assessment methods and uses standardized data extraction forms and reference management software to ensure transparency and rigor of the review process.

The scope of this review was restricted to articles published in English from 2019 to 2024 across four specific databases, excluding other databases and grey literature. This approach may have excluded relevant studies published before 2019, those not indexed in the selected databases, or those published in other languages. Additionally, only free-text articles were included, potentially introducing selection bias and limiting the comprehensiveness of the systematic review. The review findings were also limited by the heterogeneity of the included studies and differences in pharmacological approaches. The studies varied in terms of participant characteristics, dosage, and imitation protocols for platelet inhibitors following ACS. The review did not provide clarity on the risks associated with GIB, and there was a notable variation in the duration of follow-up among the studies, which could have resulted in inconsistencies in the conclusions made. Based on the results of this review, it is recommended that future research incorporate randomized controlled trials (RCTs) and observational studies with increased sample sizes and longer follow-up durations. Additional research is necessary to identify the P2Y12 inhibitor in DAPT that provides the most significant benefits for these patients.

## Conclusions

In conclusion, third-generation P2Y12 inhibitors offer cardiovascular benefits; however, the risk of GIB requires careful patient selection and monitoring. Future research should concentrate on standardizing bleeding definitions and evaluating long-term outcomes. The findings of this study indicate that ticagrelor and prasugrel may be more effective and safer than clopidogrel in ACS patients undergoing either invasive or non-invasive management. Given the significant risk of GIB, particularly in older patients or in those with a history of stroke, it is suggested to use a lower dose of prasugrel, which does not raise the risk of bleeding. In contrast to clopidogrel, patients on the novel antiplatelet regimen are relatively few in number; therefore, future clinical trials focused on an inclusive patient population using this novel antiplatelet regimen and comparing its effects with clopidogrel are needed.
